# Unraveling Natalizumab Effects on Deregulated miR-17 Expression in CD4^+^ T Cells of Patients with Relapsing-Remitting Multiple Sclerosis

**DOI:** 10.1155/2014/897249

**Published:** 2014-05-12

**Authors:** Maria Meira, Claudia Sievers, Francine Hoffmann, Maria Rasenack, Jens Kuhle, Tobias Derfuss, Ludwig Kappos, Raija L. P. Lindberg

**Affiliations:** Clinical Neuroimmunology, Departments of Biomedicine and Neurology, University Hospital Basel, Hebelstrasse 20, 4031 Basel, Switzerland

## Abstract

MicroRNAs (miRNAs) are a family of noncoding RNAs that play critical roles in the posttranscriptional regulation of gene expression. Accumulating evidence supports their involvement in the pathogenesis of multiple sclerosis (MS). Here, we compare miR-17 expressions in CD4^+^ T cells from relapsing-remitting (RR) MS patients treated with natalizumab versus untreated patients. miR-17 was downregulated under natalizumab treatment and upregulated during relapse, therefore supporting a possible role of miR-17 in MS immunopathogenesis. Downregulation of miR-17 was associated with upregulation of PTEN, BIM, E2F1, and p21 target genes. *In vitro* miR-17 inhibition was associated with upregulation of the same targets and resulted in impaired CD4^+^ T cell activation and proliferation. We further describe deregulated TGFBR2 expression in untreated patients versus healthy volunteers (HVs) and confirm *in vitro* the link between miR-17 and TGFBR2 expressions. These findings support an effect of natalizumab on expression of specific miRNA and subsequent expression of genes involved in proliferation and control of the cell cycle.

## 1. Introduction

Multiple sclerosis is a chronic inflammatory and neurodegenerative disorder of the central nervous system characterized by immune-mediated demyelination and axonal injury [1]. The heterogeneity of clinical manifestations, temporal course, and response to treatment reflects the high complexity of the illness. Although the etiology of this disabling disease is currently not yet conclusively known, MS is thought to result from an intricate interaction of genetic, viral, and environmental factors [2]. A number of transcriptomic studies aiming at defining gene expression signatures and prognostic markers have provided valuable information about the molecular mechanisms underlying MS pathogenesis [3–5]. There is growing interest in the contribution of microRNA (miRNA) expression in gene regulation of MS.

miRNAs are newly identified small (*≈*20–25 nucleotides) noncoding RNAs that mediate posttranscriptional gene silencing by binding to complementary sites on the 3′-untranslated regions (3′-UTR) of target mRNAs and by inducing mRNA cleavage or translational repression [6, 7]. miRNAs have emerged as an abundant highly conserved class of RNAs. There are about 1500 miRNAs within the human genome that are thought to regulate more than 30 percent of target genes involved in important cellular processes such as development, cell proliferation, apoptosis, and differentiation [8–11]. miRNAs are also important regulators of the innate and acquired immune responses [12]. The miR-17-92 cluster, which comprises six miRNAs (miR-17, miR-18a, miR-19a, miR-20a, miR-19b-1, and miR-92a-1), has been described as a regulator of B cell, T cell, and monocyte development [13–15].

Aberrant expression of miRNAs has been extensively investigated in various pathologies, mostly in cancer and also in MS where miRNA deregulation has been found in blood cells, in brain lesions, and in biological fluids [16–18]. Although there is increasing evidence for the involvement of B cells [19], MS pathogenesis is largely thought to be a predominant T cell mediated process [20]. Consequently, several studies have focused on miRNA dysfunction in T lymphocytes. For instance, in regulatory T cells (Tregs) of RRMS patients, De Santis et al. [21] showed specific upregulation of miR-106b and miR-25 which have been described to modulate TGF*β* signaling through their action on p21, a potent cyclin kinase inhibitor, and BIM, a Bcl-2 homology domain, proapoptotic member of the Bcl-2 family. Our previous low-density array and RT-PCR analyses provided evidence of distinct miRNA expression profiles not only in CD4^+^ T cells but also in B lymphocytes and CD8^+^ T cells from peripheral blood of RRMS patients as compared to healthy volunteers [22]. Of particular interest, miR-17 expression was upregulated in CD4^+^ T cells of untreated RRMS patients. Recently, in another study focused on miRNA expression profiles in B lymphocytes [23] we found downregulation of forty-nine miRNAs in untreated RRMS patients versus HVs. Noteworthy, two clusters, that is, miR-106b-25 and miR-17-92, were differentially expressed in patients treated with natalizumab (Tysabri, Biogen Idec and Elan Pharmaceuticals), an approved disease-modifying therapy for relapsing-remitting MS [24, 25].

Natalizumab is a recombinant humanized monoclonal antibody which binds to *α*_4_ *β*_1_ and *α*_4_ *β*_7_ integrins (also known as very late antigen-4 (VLA-4) or CD49d) on the surface of leucocytes and blocks its interaction with vascular cell adhesion molecules-1 (VCAM-1) that are expressed on the surface of endothelial cells on the lumen of blood vessels. As a selective adhesion molecule inhibitor, natalizumab impairs leucocyte adhesion and transmigration across the blood brain barrier into the central nervous system, thereby reducing inflammation and preventing the formation of new acute lesions depicted by MRI [26, 27], reducing annualized relapse rates and disability progression [28, 29]. A longitudinal gene expression profile analysis in peripheral blood of MS patients treated with natalizumab for more than 2 years revealed altered transcriptional expression of several genes related to immune response, signal transduction, adhesion, and metabolism [30]. However, little is known about natalizumab effects on posttranscriptional regulation of gene expression, particularly in T cells.

In the present study, we focus on the effect of natalizumab treatment on miR-17 expression* ex vivo* by comparing miR-17 expressions in CD4^+^ T cells from natalizumab treated* versus* untreated RRMS patients and in* in vitro* natalizumab-induced experiments. PI3K signaling is one of the potential targeted pathways of miR-17; therefore, we also study the effect of natalizumab treatment on the expression of downstream key molecules such as PTEN, an inhibitor of PI3K, BIM, and E2F1, a transcription factor that is involved in cell cycle control and regulates BIM expression [31]. Furthermore, we investigate the expression of additional potential miR-17 targets, namely, p21 [21] and TGFBR2 [32], that are involved in TGF*β* signaling, known to be impaired in MS patients. Interestingly, miR-17-92 cluster has been previously shown to be important for the regulation of internal cell processes such as cell survival and proliferation* versus* apoptosis [14]. Here, we specifically investigate the role of miR-17 in CD4^+^ T cell proliferation and activation.

## 2. Material and Methods

### 2.1. Subjects

After informed consent, fourteen untreated, fourteen natalizumab treated (mean time of treatment duration: 18 months) relapsing-remitting MS patients, and fourteen age and gender matched healthy volunteers were included in the study. All untreated patients had no immunomodulatory or other MS-specific treatments in the six months before and during the study. All natalizumab treated patients were responders to the treatment as assessed by clinical parameters, for example, had stable or improved expanded disability status scale (EDSS) and were relapse free or had a lower annualized relapse rate (RR) as compared to pretreatment. Longitudinal samples from two additional patients, who relapsed on natalizumab treatment, were obtained at the time before relapse, on relapse (before steroid therapy), and after relapse (>3 months after steroid therapy). Patient 1 (male, 31 y) was on natalizumab treatment for 3 years with an EDSS score of 4,5 resulting from a spastic-ataxic paraparesis. He experienced a relapse with a worsening of the paraparesis resulting in an EDSS score of 6,0. Patient 2 (male, 44 y) was on natalizumab treatment for 3 years with an EDSS score of 3,0 due to mild paresis, dysmetria, neurovegetative symptoms, depression, fatigue, and cognitive problems. He experienced a relapse with new onset vertigo resulting in a worsening of the cerebellar functional system score. Patient characteristics and clinical data are presented in Table 1. The Cantonal Institutional Review Board of Basel City and Basel Country approved the study.

### 2.2. Isolation of CD4^+^ T Cells

Peripheral blood mononuclear cells (PBMCs) were isolated from EDTA anticoagulated venous blood by density gradient centrifugation (Lymphoprep; Axon Lab, Switzerland). For* in vitro* studies, natalizumab (20 *μ*g/mL) was added to stimulated PBMCs in culture; CD4^+^ T cells were subsequently isolated after 24 h and processed for FACS staining and RNA isolation. The CD4^+^ T cell subpopulation was separated from PBMCs using MACS technology (CD4 MicroBeads, human; Miltenyi Biotec GmbH, Bergisch Gladbach, Germany) according to manufacturer's recommendations. The purity of isolated cells (>95%, data not shown) was measured using FITC CD3 (clone MEM-57; Immunotools, Germany) and PerCP/Cy5.5 CD4 (clone OKT4; Biolegend USA) antibodies and analyzed with an Attune Focusing Flow Cytometer (Applied Biosystems; Darmstadt, Germany). PE-anti-CD49d (clone 9F10; Biolegend USA) was used to measure CD49d surface expression.

### 2.3. RNA Isolation

Isolated CD4^+^ T cells were lysed in QIAzol (QIAgen AG, Hombrechtikon, Switzerland). Total RNA including microRNAs was extracted using miRNeasy Mini Kit (QIAgen) according to the manufacturer's protocol.

### 2.4. miRNA/mRNA Expression Analysis

Expression of miRNAs was assessed using single miRNA assays for real-time RT-PCR (Applied Biosystems; Rotkreuz, Switzerland). Megaplex Primer Pool A v2.1 (Applied Biosystems) was used for RT-PCR according to manufacturer's instructions. A preamplification step using PreAmp Primer Pool A v.2.1 (Applied Biosystems) was added to increase sensitivity. RNU44 was previously found to be the most stable control miRNA in such experimental setup and was therefore used as a reference miRNA for normalization and relative expression calculations [22]. Expression of the potential target genes was analyzed with quantitative real-time RT-PCR by using Assay-on-Demand reagents (Applied Biosystems). PUM1 was previously found to be the most stable endogenous control mRNA and was therefore used as a reference gene to normalize expression levels of target mRNAs [22]. Relative quantitation of all targets was calculated by the comparative cycle threshold method outlined in user bulletin number 2 provided by Applied Biosystems. qPCR probe sequences are described in Supplementary Table 1 (see Supplementary Table 1 in the Supplementary Materials available online at http://dx.doi.org/10.1155/2014/897249).

### 2.5. Target Prediction 

Target Scan (http://www.targetscan.org/) tools were used to predict interactions between expressed miRNAs and their potential target mRNAs.

### 2.6. miRNA Inhibition Experiments

Hsa-miR-17-5p mirVana miRNA inhibitor (sequence: CAAAGUGCUUACAGUGCAGGUAG) (Ambion Life Technologies, Switzerland) (200 pmol) or vehicle control (PBS: mock) was transiently transfected by electroporation into freshly isolated CD4^+^ T cells from healthy volunteers using the Amaxa Nucleofector system (program U-014 for high viability) and the Human T Cell Nucleofector Kit (Lonza; Köln, Germany) following the manufacturer's instructions. After resting for 4 hours to allow recovery, cells were cultured in RPMI media 1640 (Gibco, Life Technologies) supplemented with fetal bovine serum (Gibco, Life Technologies) (10%) and Penicillin/Streptomycin (Sigma-Aldrich; Switzerland) for 24 hours (for RNA isolation) or 72 hours (for proliferation/activation experiments). Mock electroporation did not significantly affect miR-17 expression. Also, a reduction in cell viability (*≈*40%) was measured by flow cytometry using LIVE/DEAD Fixable Aqua Dead Cell Stain Kit (Life Technologies) (data not shown). Inhibition efficiency was monitored by real-time RT-PCR.

### 2.7. Cell Stimulation, Protein Extraction, and Immunoblotting

PBMCs or isolated CD4^+^ T cells were stimulated with Dynabeads Human T-Activator CD3/CD28 (Invitrogen, Life Technologies) (1 : 10 or 1 : 1 bead : cell ratio) for, respectively, 24 hours (RNA isolation/FACS staining) or 48 hours (protein analysis). Nonstimulated cells served as controls. For protein extraction, cells were harvested and washed with 1x PBS and extracted using cell lysis buffer (Cell Signaling, USA) according to the procedures provided by the manufacturer. A Bio-Rad assay was performed to determine protein concentrations of samples (Bio-Rad, Hercules, CA, USA). Protein lysates were separated via SDS-PAGE and then immunoblotted onto pure nitrocellulose membranes (Bio-Rad) and probed with specific antibodies: PI3 K p85 (Cell Signaling), PTEN (Cell Signaling), Phospho-PTEN (Ser380/Thr382/383) (Cell Signaling), and Actin (C-11) (Santa Cruz Biotechnology, USA). Secondary antibodies alexa fluor 680 donkey anti-rabbit IgG (Invitrogen) and donkey anti-goat IRDye 800CW (LI-COR Biosciences, Germany) were used. Proteins were detected using the Odyssey Imaging System (LI-COR Biosciences).

### 2.8. Proliferation and Activation Assays

For proliferation assays, freshly isolated and/or rested after electroporation CD4^+^ T cells were labeled with 1 *μ*M cell trace violet cell proliferation dye (Invitrogen, Life Technologies) following the manufacturer's recommendations. Labeled cells were stimulated with Dynabeads Human T-Activator CD3/CD28 (Invitrogen, Life Technologies) (1 : 1 or 1 : 10 bead : cell ratio) for 72 hours. Cells were then harvested, stained with Live/Dead Fixable Aqua Stain (Invitrogen, Life Technologies) and processed for FACS analysis using the Attune Focusing Flow Cytometer. PE anti-human CD25 (clone BC96; Biolegend, USA) and PerCP/Cy5.5 anti-human CD4 antibody (clone OKT4; Biolegend, USA) were used to evaluate CD4^+^ T cell activation. All FACS data were analyzed with FlowJo software (Tree Star, Inc., OR, USA).

### 2.9. Data and Statistical Analysis

GraphPad Prism software (La Jolla, CA, USA) was used for all statistical analyses. Kolmogorov-Smirnov test was applied to assess normality of the data sets. One-way ANOVA with Tukey's multiple comparison test was applied to evaluate significance between various subject groups (HVs and untreated and natalizumab treated patients). Unpaired *t*-test was used to evaluate significance between two group comparisons. Paired *t*-test was used for proliferation/activation and inhibition experiments. The data were considered significant at *P* < 0.05.

## 3. Results

### 3.1. miR-17 Expression in Untreated and Natalizumab Treated RRMS Patients

**Figure 1:**
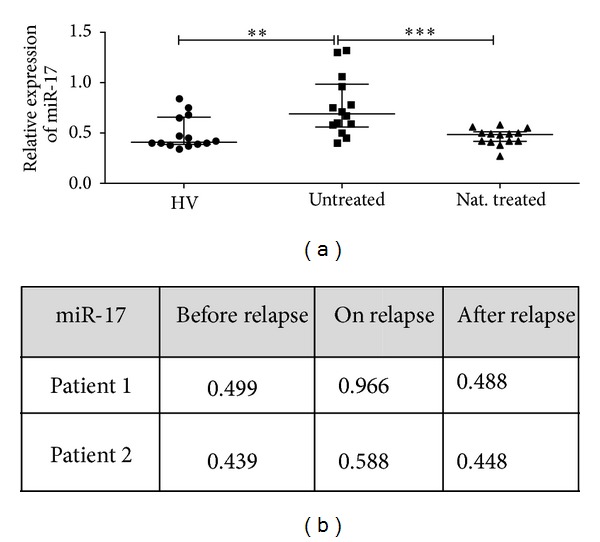
miR-17 differential expression in CD4^+^ T cells of HV and RRMS patients. Transcriptional expression of miR-17 was analyzed with real-time RT-PCR in CD4^+^ T cells from (a) HVs and untreated and natalizumab treated patients (*n* = 14) (bars represent median with interquartile range); (b) two patients on natalizumab therapy who experienced relapse (before, on, and after relapse time points). Relative expression levels are depicted. One-way ANOVA with Tukey's multiple comparison test was applied.  ***P < 0.001;  **P < 0.01.

miR-17 expression has been previously shown to be differentially upregulated in CD4^+^ T cells from untreated RRMS patients compared to HVs [22]. In the current study using another cohort of untreated RRMS patients and HV (*n* = 14), we validated upregulation of miR-17 expression in RRMS patients (1.5-fold increased expression) (*P* < 0.01, Figure 1(a)). We further analyzed relative expression levels of miR-17 in natalizumab treated patients as compared with untreated RRMS. Interestingly, natalizumab treatment decreased miR-17 expression in MS patients (1.6-fold) (*P* < 0.001, Figure 1(a)). Also, we examined expression levels of miR-17 in two patients experiencing a relapse during natalizumab therapy (Figure 1(b)). MiR-17 expression was found to be upregulated during relapse, which was reversed after the relapse.

### 3.2. Effect of Natalizumab on miR-17 Expression  *Ex* *Vivo*

**Figure 2:**
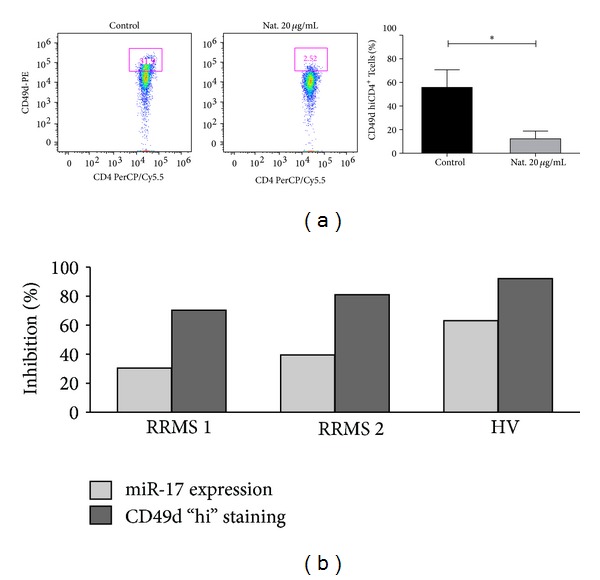
Natalizumab effect* ex vivo*. Isolated CD4^+^ T cells from untreated (control) or natalizumab treated PBMCs were stained for CD49d expression and processed for miR-17 expression quantification (RRMS, *n* = 2; HV, *n* = 1). (a) (Left panel) Representative FACS dot plots of CD4 and CD49d surface expression in cells from a HV. (Right panel) Percentage of CD49d hi cells among CD4^+^ T cell populations. Bars represent mean with SEM (standard error of the mean) in three independent experiments; (RRMS, *n* = 2; HV, *n* = 1). (b) Percentage of inhibition of miR-17 expression and CD49d hi staining in natalizumab treated cells. Paired *t*-test was applied.  *P < 0.05.

We examined* ex vivo* the effect of natalizumab on miR-17 expression in CD4^+^ T cells isolated from stimulated RRMS (*n* = 2) and HV-derived (*n* = 1) PBMCs. Natalizumab significantly reduced CD49d hi surface expression (Figure 2(a)). Furthermore, expression of miR-17 was downregulated in natalizumab treated cells in the three independent experiments (Figure 2(b)). Notably, increased inhibition of CD49d hi staining was associated with decreased expression of miR-17 (Figure 2(b)), suggesting a potential direct effect of natalizumab on miR-17 expression levels in CD4^+^ T cells.

### 3.3. Expression of miR-17 Target mRNAs

**Figure 3:**
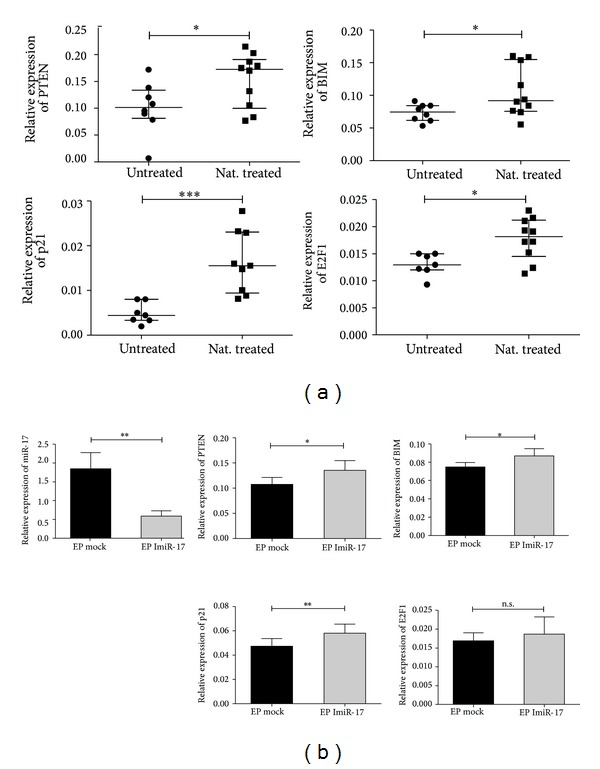
Expression of candidate target mRNAs. (a) Transcriptional expressions of PTEN, BIM, p21, and E2F1 were analyzed with real-time RT-PCR in CD4^+^ T cells from untreated and natalizumab treated patients (untreated: *n* = 8; Nat. treated: *n* = 10) (bars represent median with interquartile range) and (b) in freshly isolated CD4^+^ T cells from HVs transiently transfected by electroporation (EP) with 1 *μ*M of miR-17 synthetic inhibitor (ImiR-17) or vehicle control (mock) ((b) middle and right panels). Analysis of miR-17 transcriptional expression ((b) left panel). Values represent mean with SEM for 8 independent experiments. Relative expression levels are depicted. Unpaired two-tailed *t*-test was used.  ***P < 0.001;  **P < 0.01;  *P < 0.05; n.s.: not significant.

We analyzed the effect of natalizumab on the expression of potential miR-17 targets. Interestingly and in accordance with the downregulation of miR-17 expression under natalizumab therapy, PTEN and BIM mRNA expression levels were upregulated in natalizumab treated (*n* = 10) compared to untreated patients (*n* = 8) (1.5- and 1.4-fold, resp.) (Figure 3(a) top panels). Also, significant upregulation of p21 and E2F1, both predicted miR-17 target mRNAs, was found in natalizumab treated patients (3.3-fold for p21 and 1.3-fold for E2F1) (Figure 3(a), bottom panels). To study the* in vitro* effect of miR-17 inhibition on the expression of the analyzed potential targets* ex vivo*, we performed transfection experiments of isolated CD4^+^ T cells with a synthetic inhibitor of miR-17. Efficient and significant inhibition was measured after 24 h (Figure 3(b) left panel). Specific inhibition of miR-17 resulted in upregulation of all four target mRNAs, with achieved significance for PTEN, BIM, and p21 (Figure 3(b) middle and right panels).

### 3.4. Deregulated Expression of TGFBR2 in MS

**Figure 4:**
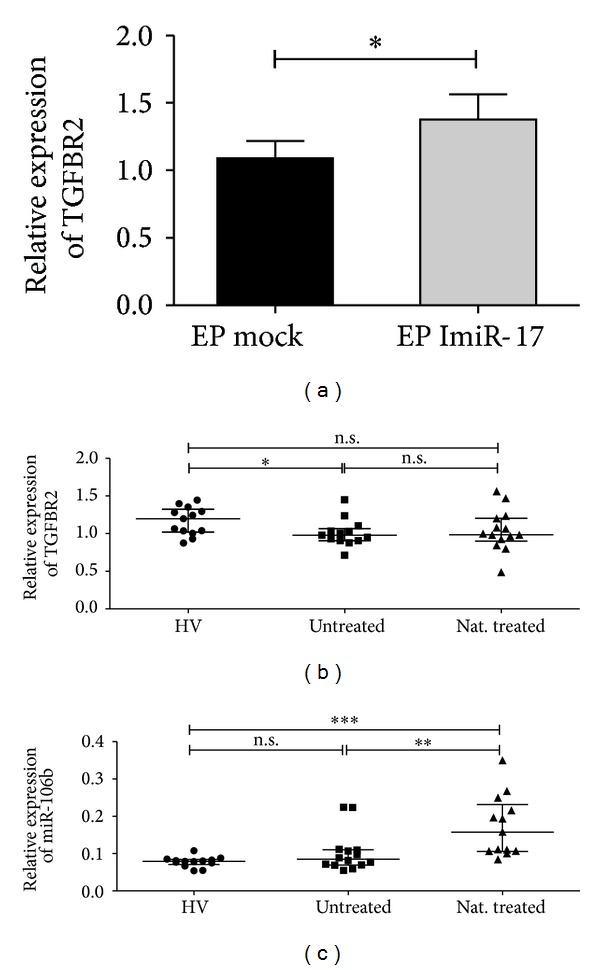
TGFBR2 and miR-106b expression analysis. (a) Transcriptional expression of TGFBR2 was analyzed with real-time RT-PCR in freshly isolated CD4^+^ T cells from HVs transiently transfected by electroporation (EP) with 1 *μ*M of miR-17 synthetic inhibitor (ImiR-17) or vehicle control (mock) (*n* = 7) (bars represent mean with SEM) and (b) in CD4^+^ T cells from HVs and untreated and natalizumab treated patients (*n* = 14). (c) Analysis of transcriptional expression of miR-106b in CD4^+^ T cells from HVs and untreated and natalizumab treated patients (*n* = 14). Bars represent median with interquartile range. Relative expression levels are depicted. One-way ANOVA with Tukey's multiple comparison test was applied.  ***P < 0.001,  **P < 0.01;  *P < 0.05; n.s.: not significant.

In our experimental setup, specific inhibition of miR-17 in purified CD4^+^ T cells resulted in a significant upregulation of TGFBR2 expression (Figure 4(a)), therefore corroborating a link between miR-17 and TGFBR2 expression. Moreover, the analysis of TGFBR2 expression* ex vivo* revealed a significant downregulation of TGFBR2 expression in RRMS patients compared to HVs (Figure 4(b)), reflecting a possible direct association with the reported miR-17 upregulation in MS. However, while miR-17 expression appears to be downregulated in natalizumab treated versus untreated patients, no significant expected upregulation of TGFBR2 was found in CD4^+^ T cells from natalizumab treated patients (Figure 4(b)), suggesting that mechanisms other than the transcriptional repression of TGFBR2 by miR-17 are likely involved. Considering that multiple miRNAs can regulate the expression of a single target, we examined other potential regulators of TGFBR2 expression. Interestingly, miR-106b has been found to directly target TGFBR2 and enhance iPS cell generation [33]. In the current study and in accordance with our previous low-density array analysis [22], no significant change was found for miR-106b expression in RRMS compared to HVs (Figure 4(c)). However, miR-106b was significantly upregulated in CD4^+^ T cells from natalizumab treated patients (Figure 4(c)), suggesting a possible inverted effect of both miR-17 and miR-106b on TGFBR2 expression.

### 3.5. Effect of miR-17 on CD4^+^ T Activation and Proliferation

**Figure 5:**
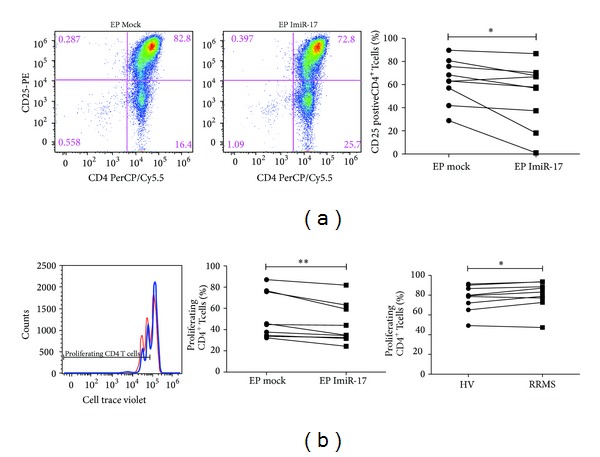
Flow cytometric analysis of CD4^+^ T cell activation and proliferation. (a) CD4^+^ T cell activation. (Left panel) Representative FACS dot plots of CD4 and CD25 surface expression in CD3/CD28 (1 : 10 bead/cell ratio) stimulated CD4^+^ T cells from a HV following electroporation (EP) with a miR-17 synthetic inhibitor (ImiR-17) or vehicle control (mock). The percentage of cells contained within each subset is indicated. (Right panel) Frequency of CD4^+^CD25^+^T cells in 9 independent miRNA-17 inhibition experiments. (b) CD4^+^ T cell proliferation. CD4^+^ T cells were labeled with cell trace violet proliferation dye and then placed in culture in the presence of CD3/CD28 stimulus for 72H and examined by flow cytometry. (Left panel) Representative histogram plot of cell proliferation in CD3/CD28 (1 : 10 bead/cell ratio) stimulated CD4^+^ T cells from a HV following electroporation (EP) with a miR-17 synthetic inhibitor (blue line) or vehicle control (mock) (red line). Proliferating CD4^+^T cells are depicted. (Middle panel) Frequency of proliferating CD4^+^ T cells in 9 independent miRNA-17 inhibition experiments. (Right panel) Frequency of proliferating CD4^+^ T cells from HVs versus RRMS untreated patients following CD3/CD28 stimulation (1 : 1 bead/cell ratio) (*n* = 9). Paired *t*-test was used.  **P < 0.01;  *P < 0.05.

We investigated a potential role of miR-17 on CD4^+^ T cell activation. To partially mimic physiological stimulation by antigen-presenting cells [34], CD3/CD28 Dynabeads were used to activate CD4^+^ T cells. Interestingly, miR-17 specific inhibition reduced CD25 expression on activated cells* in vitro* (Figure 5(a)), favoring a positive effect of miR-17 on lymphocyte activation.

We further analyzed the role of miR-17 on CD4^+^ T cell proliferation. Specific inhibition of miR-17 in CD4^+^ T cells resulted in a significant impairment of cell proliferation upon* in vitro* CD3/CD28 stimulation (Figure 5(b) left and middle panels), suggesting an important role of miR-17 on lymphocyte proliferation. Also and in accordance with previously described miR-17 upregulation in RRMS patients [22], the analysis of CD4^+^ T cell proliferation upon* in vitro* stimulation revealed a significant increase in the proliferating capacity of lymphocytes from untreated RRMS patients compared to HVs (Figure 5(b) right panel).

## 4. Discussion

Growing evidence on deregulation of miRNAs in MS has suggested their critical regulatory role in the pathogenesis of the disease [35]. So far, very few studies have investigated the impact of MS therapies on dysregulated miRNAs. Waschbisch et al. [36] showed that deregulation of miR-146a and miR-142-3p expression in PBMCs was normalized by treatment with Glatiramer Acetate. More recently, our group identified specific miRNA expression profiles in B lymphocytes from natalizumab treated patients, therefore suggesting that natalizumab could mediate some effects through miRNA regulation [23]. Of particular interest, natalizumab treatment “reversed” deregulated expression of members of both miR-17-92 and miR-106b-25 clusters, two paralogs whose functional cooperation was shown in mice [13], back to the levels of HV in B cells. In the present study using CD4^+^ T cells, we showed that natalizumab “normalized” deregulated miR-17 expression and induced significant upregulation of miR-106b, whereas, in line with our previous low-density array analysis, no difference was found between untreated RRMS patients and HVs [22]. Thus, natalizumab has an inverse impact on the expression of miR-17 and miR-106b in CD4^+^ T cells, possibly reflecting overlapping or compensation mechanisms upon treatment. Also, the reported rapid reduction in disease activity under natalizumab therapy [28] is in line with the kinetics of miR-17 downregulation that was also seen within the first 3 months (data not shown), suggesting that natalizumab-induced miRNA regulation has a role already in the early beneficial therapeutic effects of natalizumab.

The miR-17-92 and miR-106b-25 clusters have emerged as important regulators of transforming growth factor *β* (TGF*β* signaling) [37]. While miR-17 was found to modulate CD4^+^ T cell effector responses by targeting TGFBR2 [38], the same target was also shown to be regulated by miR-106b [33]. In the current study, loss of function experiments using a miR-17 inhibitor confirmed the link between miR-17 and TGFBR2 mRNA expression. Interestingly, in accordance with the miR-17 upregulation in untreated RRMS patients, we found significant downregulation of TGFBR2 mRNA, suggesting miR-17 dependent regulation of the TGF*β* pathway. However, no significant change of TGFBR2 mRNA expression was found in cells from patients under natalizumab therapy, reflecting a possible interrelating regulation by miR-106b. These are interesting findings regarding the pivotal role of TGF*β* in the immune system [39]. Indeed, Li and colleagues showed that T cell-specific deletion of TGFBR2 resulted in lethal inflammation associated with T cell activation and differentiation in mice [40]. Importantly, the TGF*β* pathway regulates maintenance of peripheral Foxp3-expressing regulatory T cells [41], whose frequency and immunosuppressive function were shown to be reduced in the course of MS [42, 43]. Considering the reported suppressive role of miR-17 on iTreg differentiation [38], it appears essential to further study the miR-17 regulation of Treg differentiation in MS.

We further investigated the expression of two other potential target genes of miR-17 that are also known to be effectors of TGF*β* signaling [44, 45], namely, the cyclin-dependent kinase inhibitor 1, p21, and a proapoptotic member of the Bcl-2 family, BIM, [46]. In line with the downregulation of miR-17, both transcripts were significantly upregulated in CD4^+^ T cells of natalizumab treated compared to untreated patients, suggesting that natalizumab could affect mechanisms of cell cycle progression and apoptosis through miR-17 regulation. Interestingly, Petrocca and colleagues showed that upregulation of the miR-106b-25 cluster also impairs the TGF*β* pathway by interfering with the expression of the same targets p21 and BIM [47]. In addition, E2F1, a transcription factor that controls the G1-S transition, was shown to activate the miR-106b-25 cluster, which in turn negatively regulates E2F1 expression [47]. In parallel, O'Donnell and colleagues showed that c-myc activates the expressions of the miR-17-92 cluster and E2F1, which is itself also a target of miR-17 [48], therefore making the entire regulatory network very complex. In our study, we showed that E2F1 mRNA expression was upregulated in natalizumab treated patients, favoring more the model of a miR-17-mediated regulation. However, regarding the complexity of the miRNA network, we cannot exclude the contribution of other members of these two clusters that regulate same genes.

The pathway analysis of potential target genes of miR-17 also revealed the PI3 K/Akt pathway, described as a critical regulator of T cell development [49], as one of the most represented. Consistent with the reported CD3/CD28-induced upregulation of miR-17 in CD4^+^ T cells [22], PI3KR1 and PTEN mRNA expressions were strongly downregulated upon stimulation (Supplementary Figure S1A). We further analyzed PI3KR1 and PTEN protein expressions and showed specific repression of both expressions and phosphorylation of PTEN upon stimulation (Supplementary Figure S1B), which is well reflecting the downregulation of respective mRNAs. In accordance with miR-17 downregulation in natalizumab treated patients, we found upregulation of PTEN mRNA, an interesting finding regarding the important role of PTEN in the regulation of T cell homeostasis and self-tolerance [50, 51]. Interestingly, a relative decrease of CD4^+^CD25^+^ regulatory T cells has been described in natalizumab treated patients [52]. Based on the reported PTEN inhibition of IL-2 receptor mediated expansion of CD4^+^CD25^+^ Tregs [53], one could speculate that miR-17 downregulation in natalizumab treated patients would also possibly impact on Tregs frequencies through PTEN activation.

The appropriate balance between PI3 K and PTEN is necessary to maintain normal T cell responses [54]. In our previous study, we have identified PI3KR1 as a potential target of miR-17 [22]. However, in the current study, despite a trend for increased expression, we did not find significant differences in the mRNA expression of PI3KR1 between untreated and natalizumab treated patients (Supplementary Figure S1C). This might be due to small patient numbers, or it can also suggest that mechanisms other than miR-17 regulation could affect PI3KR1 expression. In fact, one miRNA can target multiple genes and conversely one gene can be regulated by several miRNAs, therefore adding to the complexity of the network analysis. It should also be mentioned that human aging was shown to affect miRNA expression patterns [55]. However, the analysis of our samples did not reveal any correlation between miR-17 expression and age (Supplementary Figure S2).

The present study highlights an important role of miR-17 in CD4^+^ T cell proliferation and activation. Importantly, the miR-17-92 cluster has also been proposed to promote cell proliferation in various studies. Xiao and colleagues generated lymphoproliferative disease and autoimmunity in mice by overexpression of the miR-17-92 cluster in lymphocytes. Suppressed expression of PTEN and BIM and enhanced CD3/CD28-induced proliferation of CD4^+^ T cells were reported in miR-17-92-transgenic mice [14]. Another study by Woods and colleagues proposed a model whereby miR-17-92 promotes cell proliferation by shifting the E2F transcriptional balance away from E2F1 toward the proliferative E2F3 transcriptional network [56]. Here, we show that miR-17 regulates the expression of these important proliferation effectors, therefore adding to our understanding of its function in the process of MS pathogenesis.

## 5. Conclusions

Our results support an involvement of miRNAs in MS immunopathogenesis and in the molecular mechanism of natalizumab therapy. The mechanism by which natalizumab influences the expression of miR-17 remains unclear although our* in vitro *experiments suggest a possible direct effect. The strong upregulation of miR-17 during relapse in patients under natalizumab treatment also supports an important role of miR-17 regulation in the pathogenesis of MS. However, our findings need to be validated in large and independent MS cohorts. Also, further longitudinal studies with additional patient samples are necessary to understand long-term effects of natalizumab as well as the mechanisms of the progression of MS.

## Supplementary Material

Supplementary material includes a list of sequences of miRNAs and mRNAs (Table 1) and figures of PI3KR1 and PTEN expression analysis (Figure S1) and correlation analysis of age with relative expression of miR-17 (Figure S2).

## Figures and Tables

**Table 1:** Characteristics of patients and healthy volunteers (HVs).

Group	Gender F/M	Age (years) Mean ± SD	Disease duration (years)** Mean ± SD	EDSS** Mean ± SD	EDSS before Nat. treatment Mean ± SD	Annualized relapse rate during last 3 years Mean ± SD	Annualized relapse rate on Nat. treatment Mean ± SD	Number of relapse free patients on Nat. treatment
HV (*n* = 14)	10/4	39.6 ± 10.1	na	na	na	na	na	na
Untreated (*n* = 14)	10/4	46.9 ± 9.4*	9.64 ± 5.77	2.07 ± 1.08	na	0.45 ± 0.82	na	na
Nat. treated (*n* = 14)	10/4	39.4 ± 8.9	8.42 ± 4.83	3.03 ± 1.51	3.53 ± 1.39	0.83 ± 0.44***	0.35 ± 0.40	6/14

na: not applicable.

*Untreated RRMS patients are older than HVs (n.s.) and natalizumab (Nat.) treated patients (*P* < 0.05).

**At the sampling time.

***Before natalizumab (Nat.) treatment.

## Abbreviations

RRMS: Relapsing-remitting multiple sclerosis
HV: Healthy volunteer
miRNA: MicroRNA
Nat.: Natalizumab
PTEN: Phosphatase and tensin homology
TGFBR2: Transforming growth factor, beta receptor II
p21/CDKN1A: Cyclin-dependent kinase inhibitor 1
iPS: Induced pluripotent stem cells
EDSS: Expanded disability status scale
Annualized RR: Annualized relapse rate
SEM: Standard error of the mean.
