# Supplementary material for: Unraveling Natalizumab Effects on Deregulated miR-17 Expression in CD4+ T Cells of Patients with Relapsing-Remitting Multiple Sclerosis
Source: J Immunol Res. 2014 May 12;2014:897249. doi: 10.1155/2014/897249 (PMC4036714; doi:10.1155/2014/897249)
Supplement: Supplementary file 1 — Supplementary material includes a list of sequences of miRNAs and mRNAs (Table 1) and figures of PI3KR1 and PTEN expression analysis (Figure S1) and correlation analysis of age with relative expression of miR-17 (Figure S2). [file 897249.f1.pdf]

Table 1. qPCR probe sequences of miRNAs and mRNAs.

Figure S1. PI3KR1 and PTEN expression analysis. (A) $CD4^+$T cells isolated from HV samples were stimulated with CD3/CD28 antibodies *in vitro* for 24 hours. Transcriptional expression of PI3KR1 (N=7) and PTEN (n=8) were analyzed with real time RT-PCR. (B) Western blot analysis of lysates from purified $CD4^+$T cells (HV) after mitogenic stimulation with CD3/CD28 antibodies and probed for PI3KR1, PTEN, and phospho-PTEN. Actin levels were monitored as a control for loading. (C) Transcriptional expression of PI3KR1 was analyzed with real-time RT-PCR in and in $CD4^+$T cells from untreated and natalizumab treated patients (untreated: n=8; Nat. treated: n=10). ***p<0.001; **p<0.01; n.s: not significant.

Figure S2. Correlation of age with relative expression of miR-17. Non-parametric Spearman correlation, with two-tailed p-value, between miR-17 expression and age in HVs, untreated and natalizumab treated RRMS patients, was calculated. 95% confidence interval is depicted by dotted curves.

miR-17	CAAAGUGCUUACAGUGCAGGUAG
miR-106b	UAAAGUGCUGACAGUGCAGAU
RNU44	CCTGGATGATGATAGCAAATGCTGACTGAACATGAAGGTCTTAATTAGCTCTAACTGACT
PTEN	CCTCTTCCTCGGCTTCTCCTGAAAG
BIM	CAGTGCAATGGCTTCCATGAGGCAG
P21	CAGCATGACAGATTTCTACCACTCC
E2F1	TCCAGTGGCTGGGCAGCCACACCAC
TGFBR2	GCTCAACCACCAGGGCATCCAGATG
PI3KR1	GGGAGATATCTCGAGGGAAGAAGTG
PUM1	CTACCGCTCCGCCCGTGTTGGTGGA

Supplementary Table 1.

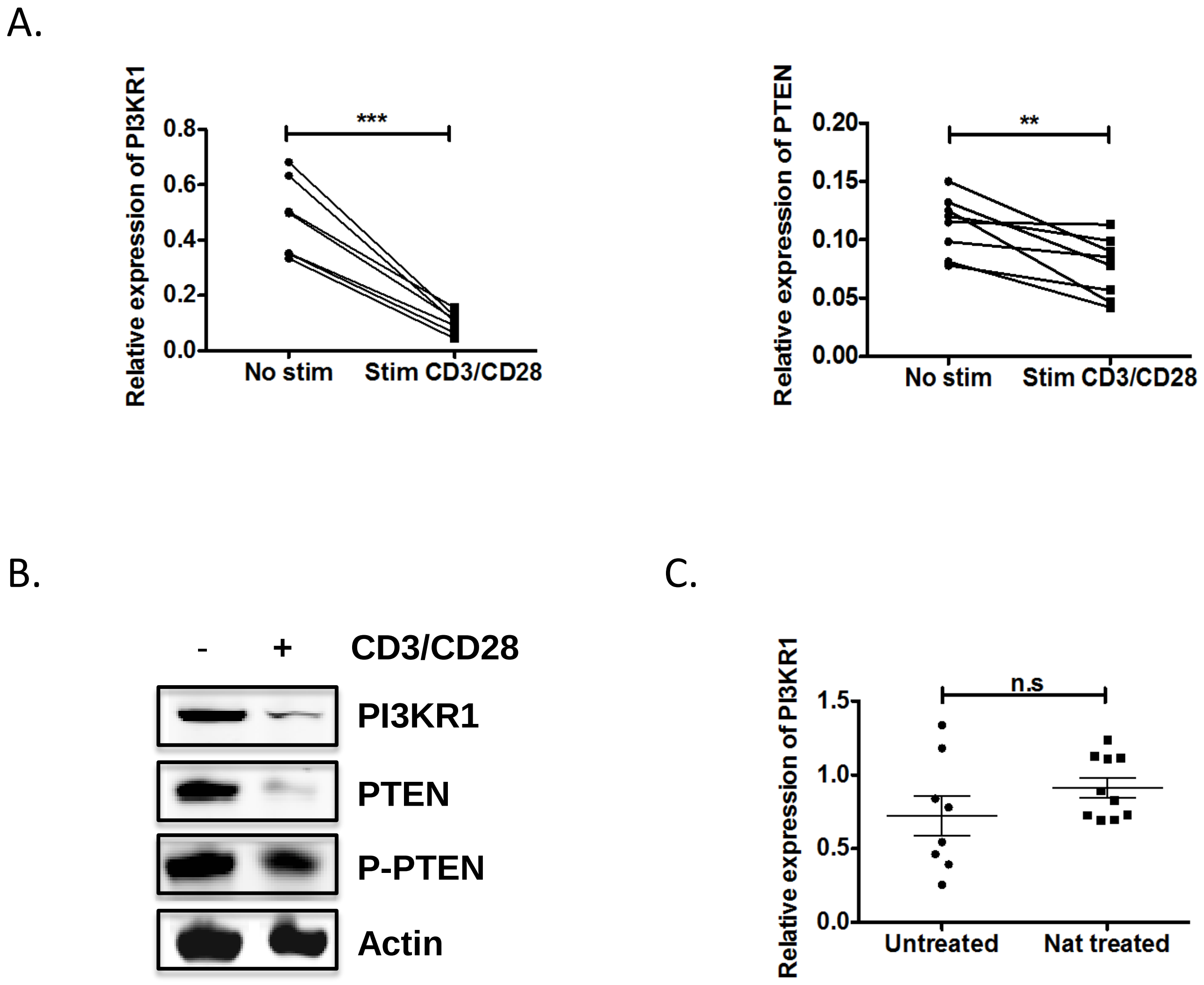

A.
Relative expression of PI3KR1
0.8
0.6
0.4
0.2
0.0
***
No stim
Stim CD3/CD28
Relative expression of PTEN
0.20
0.15
0.10
0.05
0.00
**
No stim
Stim CD3/CD28
B.
-
+
CD3/CD28
PI3KR1
PTEN
P-PTEN
Actin
C.
Relative expression of PI3KR1
1.5
1.0
0.5
0.0
n.s
Untreated
Nat treated

Figure S1.

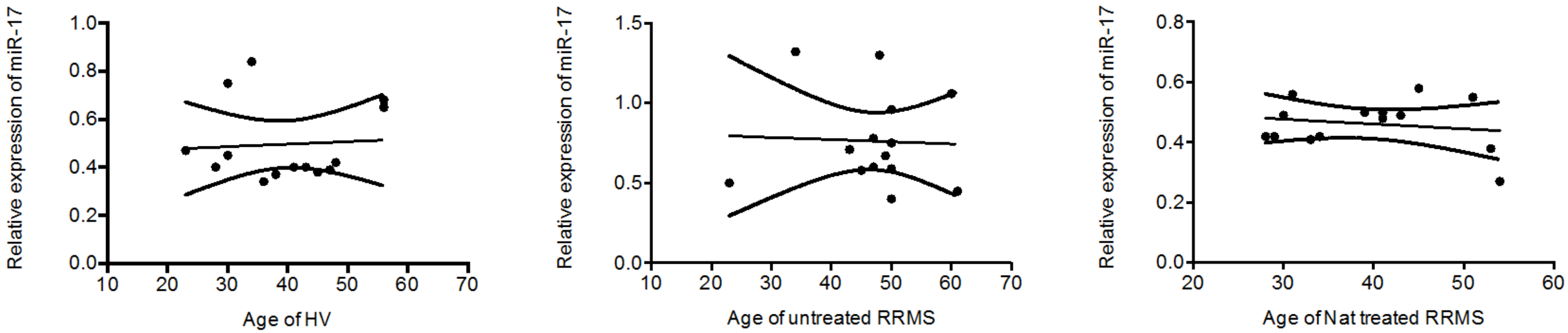

Spearman r	-0.07080
95% confidence interval	-0.5912 to 0.4912
P value (two-tailed)	0.8099
P value summary	ns

Spearman r	-0.1090
95% confidence interval	-0.6157 to 0.4614
P value (two-tailed)	0.7106
P value summary	ns

Spearman r	-0.02104
95% confidence interval	-0.5578 to 0.5281
P value (two-tailed)	0.9431
P value summary	ns

Figure S2.
